# Cross-Translational Studies in Human and *Drosophila* Identify Markers of Sleep Loss

**DOI:** 10.1371/journal.pone.0061016

**Published:** 2013-04-24

**Authors:** Matthew S. Thimgan, Laura Gottschalk, Cristina Toedebusch, Jennifer McLeland, Allan Rechtschaffen, Marcia Gilliland-Roberts, Stephen P. Duntley, Paul J. Shaw

**Affiliations:** 1 Department of Anatomy and Physiology, Washington University School of Medicine, St. Louis, Missouri, United States of America; 2 Department of Neurology, Washington University School of Medicine, St. Louis, Missouri, United States of America; 3 Department of Psychiatry, University of Chicago, Chicago, Illinois, United States of America; Hôpital du Sacré-Coeur de Montréal, Canada

## Abstract

Inadequate sleep has become endemic, which imposes a substantial burden for public health and safety. At present, there are no objective tests to determine if an individual has gone without sleep for an extended period of time. Here we describe a novel approach that takes advantage of the evolutionary conservation of sleep to identify markers of sleep loss. To begin, we demonstrate that IL-6 is increased in rats following chronic total sleep deprivation and in humans following 30 h of waking. Discovery experiments were then conducted on saliva taken from sleep-deprived human subjects to identify candidate markers. Given the relationship between sleep and immunity, we used Human Inflammation Low Density Arrays to screen saliva for novel markers of sleep deprivation. *Integrin αM* (*ITGAM*) and *Anaxin A3* (*AnxA3*) were significantly elevated following 30 h of sleep loss. To confirm these results, we used QPCR to evaluate *ITGAM* and *AnxA3* in independent samples collected after 24 h of waking; both transcripts were increased. The behavior of these markers was then evaluated further using the power of *Drosophila* genetics as a cost-effective means to determine whether the marker is associated with vulnerability to sleep loss or other confounding factors (e.g., stress). Transcript profiling in flies indicated that the *Drosophila* homologues of *ITGAM* were not predictive of sleep loss. Thus, we examined transcript levels of additional members of the integrin family in flies. Only transcript levels of *scab*, the *Drosophila* homologue of *Integrin α5* (*ITGA5*), were associated with vulnerability to extended waking. Since *ITGA5* was not included on the Low Density Array, we returned to human samples and found that *ITGA5* transcript levels were increased following sleep deprivation. These cross-translational data indicate that fly and human discovery experiments are mutually reinforcing and can be used interchangeably to identify candidate biomarkers of sleep loss.

## Introduction

During the last decade it has become clear that insufficient sleep increases the susceptibility of individuals to serious diseases, including obesity, type II diabetes and coronary heart disease to name but a few [1]–[3]. Moreover, sleep loss results in substantial deficits in cognition such that inadequate sleep is considered to be a causal factor in many occupational and motor vehicle accidents [4], [5]. Indeed, sleep deprivation can impair performance to a similar level as that observed in subjects with a blood alcohol concentration of 0.10% [6]. Taken together with the observation that 20–40% of adults sleep less than 7 h/night [7], these data indicate that inadequate sleep imposes a substantial burden on public health and safety, and that efforts to mitigate these negative outcomes would be facilitated if an objective method to identify sleep-deprived individuals was available[5], [8].

**Figure 1:**
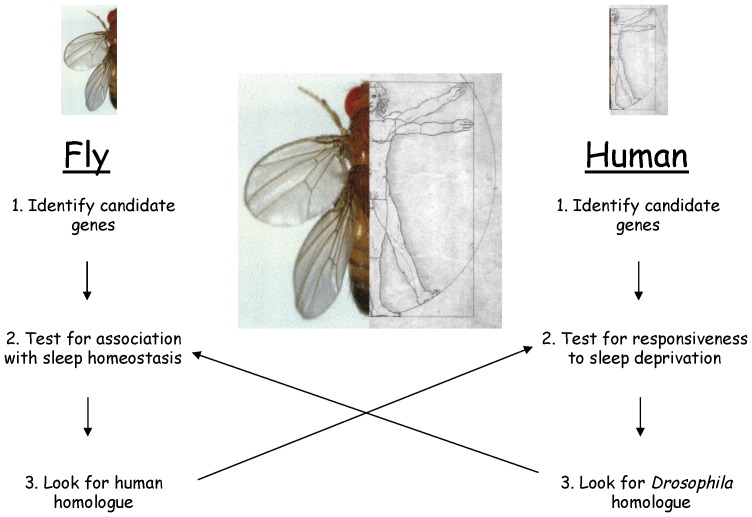
Overall strategy to exploit the evolutionary conservation of sleep to identify and validate biomarkers of sleepiness.

Using the power of *Drosophila* genetics, we have identified transcripts, including *Amylase*, that are tightly linked with sleep homeostasis in the fly but are unchanged when episodes of extended-waking are not accompanied with subsequent increases in sleep (sleep rebound) [9]. Follow-up translational studies revealed that *Amylase* levels are also significantly increased in human saliva following a night of sleep loss [9]. The increase in *Amylase* could not be attributed to stress in either the fly or the human. Consistent with this observation, humans with reduced *adenosine deaminase* activity show enhanced slow wave sleep, increased feelings of being sleepy, impaired performance on the psychomotor vigilance task (PVT) and elevated levels of salivary *Amylase* activity [10]. Similarly to *Amylase*, transcript levels of *Filamin-A*, *Malic enzyme* and *TSC22D* were first identified as responsive to sleep loss in flies and subsequently found to be modified in sleep deprived humans [11], [12]. Thus, the fly can be used to identify sleep-loss related transcripts as a starting point to identify salivary biomarkers of sleep loss in humans (Figure 1).

Given that genes are pleiotropic and that the genetic and environmental context of a gene can influence its behavior, it is unlikely that single transcript will be able to uniquely identify sleep deprived individuals [13]–[15]. As a consequence, an effective test of sleep loss will likely require a panel of biomarkers that both respond to increased wake time and have a different spectrum of outside influences (e.g., illness, metabolism, sympathetic tone, etc.). Since components of the human salivary transcriptome are responsive to increased wakefulness [9], [11], [12] we hypothesized that we could screen for additional candidate biomarkers of sleep loss directly in humans, and then, using a cross-translational approach, validate these candidates using the numerous genetic and pharmacologic protocols in the fly to determine if the biomarkers reflect sleep need [9], [13] (Figure 1). However, since human saliva contains as many as 3000 transcripts [16], the first question we needed to address was where should we begin? Since, immune-related molecules have been strongly implicated for their involvement in sleep regulation in both mammals and flies [17], [18], and serum inflammatory markers are highly responsive to sleep disruption[19], [20], we hypothesized that 1) mRNAs associated with inflammatory molecules would be significantly elevated in human saliva following sleep loss and 2) that when evaluated in flies, these inflammatory molecules would only be elevated in flies following episodes of waking that are accompanied by a sleep rebound.

## Materials and Methods

### Rodent Studies

#### Subjects

20 male Sprague Dawley rats, 7 rats exposed to Total Sleep Deprivation (TSD), 6 yoked controls (TSC), and 7 home cage controls (HCC). Rats weighed 464±12 g (SEM) at surgery. The rats were individually housed at 25+/−1°C under constant light for two weeks to avoid confounding subsequent sleep loss with shifts in phase, period or amplitude of circadian rhythms [21]. Food and water were available *ad libitum*.

#### Surgery

Rats were pre-treated with atropine sulfate (i.p., 0.1 mL) and anesthetized with Nembutal (i.p., 1.1 mg/kg). Surgical procedures have been previously described [22]. Briefly, two miniature screw EEG electrodes were placed through the skull at lateral sites, two screw electrodes were placed near the midline to record hippocampal “theta” activity, and stainless steel wire (Model A3632, Cooner Wire Co., Chatsworth, CA) was sutured into the nuchal muscle to record electromyogram (EMG).

#### Apparatus and Variables Measured

After one week of postoperative recovery, rats were placed into the deprivation apparatus for a 7-day baseline period consistent with previous studies [23]. TSD and TSC rats were connected by a recording cable to a commutator suspended from a counterbalanced boom that allowed them free movement throughout individual plastic cages. A single horizontal fiberglass disk (46 cm diameter) partially extended under both cages to form the floor, which was suspended over 2–3 cm deep water. Electrophysiological signals were passed sequentially from the rat to a polygraph, and to an AIM-65 microcomputer, where they were rectified and integrated. During the deprivation period, the disk was rotated when sleep onset was detected in the TSD rat, forcing both the TSD and TSC rats to move to avoid the water. However, the TSC rat could sleep when the disk was still. The PASS scoring system computer scored 30 sec epochs for wake, Rapid Eye Movement (REM) sleep and three Non-REM (NREM) sleep stages: low EEG amplitude (LS), medium EEG amplitude (HS1), and high EEG amplitude (HS2) NREM sleep. Body weight, food intake, and water intake were measured daily. Energy expenditure (EE) was calculated from the caloric value of food eaten and a running average of the caloric value of weight change (energy storage). This method has been previously validated in TSD rats [24].

#### Blood collection and Cytokine analysis

After 8 days of TSD, rats were deeply anesthetized with pentobarbital (55 mg/kg). The thoracic cavity was opened and 5 mL of blood was collected from the heart under sterile conditions. Blood culture bottles (tryptic soy broth and CO2, DIF-CO Blood Culturing System: DIFCO Laboratories, Detroit, MI) were inoculated with 3 mL of blood. The remaining 2 mL of blood was placed into chilled Vacutainer tubes (Becton- Dickinson, Franklin Lakes, NJ), centrifuged, and the serum was stored in aliquots at −70°C until the day of analysis.

Blood culture bottles were vented and incubated for 24 hrs. at 37°C, visually examined, subcultured to three media–MacConkey (for Gram-negative bacilli), CAN (for Gram-positive cocci), and blood agar plates (TSA with 5% sheep blood for Gram-positive cocci and both Gram- positive and Gram–negative bacilli) –and incubated for another 24 hours at 37°C. Microbes were identified in accordance with standard microbiological techniques using Gram stains, the Analytical Profile Index (BioMerieux Vitek, Hazelwood, MD), and other biochemical tests as needed. Negative cultures were reported after 7 days of incubation, consistent with standard procedure.

Serum concentrations of Tumor Necrosis Factor alpha (TNFα), Interleukin beta (IL-1β), Interferon gamma (INF-γ) were determined using commercial ELISA kits (Biosource, Camarillo, CA). Rat Interleukin-6 (IL-6) was quantified using mouse ELISA kit (R&D Systems, Minneapolis, MN) which has been reported to cross-react with rat IL-6. All assays were performed in duplicate. All blood work, including detection of bacteria and ELISA was performed by the RH Rust Diagnostic Laboratory (Animal Resource Center, University of Chicago).

#### Data Analysis

In evaluating the differences between TSD, TSC, and Home Cage Controls (HCC) rats, food intake, body weight, and EE was expressed as percent of baseline. All variables were evaluated by simple analysis of variance.

### Human Studies

#### Subjects

Nine healthy human adult volunteers (seven men and two women), 37±3.3 years of age with a BMI of 25±1.4 and with no history of neurological or psychiatric disease were enrolled in the study after providing their consent. The study was approved by the Institutional Review Board at Washington University School of Medicine. Sleep time was monitored for the week prior to the onset of the study using actigraphy to confirm adequate baseline sleep prior to entry into the study (Actiwatch 64, Mini Mitter Bend, OR, USA). The subjects arrived at the Washington University Sleep Laboratory at ∼7∶30 AM and were constantly monitored by two experienced, registered sleep technologists throughout the remainder of the study. Every 6 h, the Stanford Sleepiness Scale (SSS) and a 10-minute version of the Psychomotor Vigilance Test PVT [25] were administered following which saliva was collected using plain (non-citric acid) cotton Salivettes (Sarstedt, Newton, NC) with 1 mL of RNALater (Qiagen). Samples were immediately frozen on dry ice and kept at −80°C until assayed. Meals were not allowed 1 hour prior to saliva collection and caffeine intake was not permitted. Samples from our previous study (n = 8) [9] were combined with the current subjects for the evaluation of salivary IL-6.

#### Salivary IL-6

Quantification of IL-6 was carried out using ELISA designed to detect human IL-6 according to manufacturer instructions (R&D Systems, Minneapolis, MN).

#### Quantitative PCR

For preparation of cDNA from saliva, salivettes were thawed and saliva was extracted by centrifugation. RNA was isolated from cell-free supernatant as previously described [9]. Isolated RNA was reverse-transcribed by using SuperScript III (Invitrogen Life Technologies, Carlsbad, CA) according to the manufacturer's instructions. Quantitative PCR using SYBR based technology was performed by using the 7600 Real-Time PCR System (Applied Biosystems, Foster City, CA). A 9-ul aliquot of the cDNA was used in each reaction, and all reactions were performed in duplicate. For quantitation of saliva samples, CT values were set and then normalized to *β-actin* within the respective sample. Subsequently, normalized values were compared between samples. Predesigned Inflammation Low Density Arrays were purchased from Applied Biosystems Inc. (catalog # 4378707). mRNA levels were quantified at 2 pm under baseline conditions and at 2 pm the next day following 30 h of extended waking. Low Density Arrays were processed according to manufacturer's instructions.

### Fly Studies

#### Subjects

Flies were reared in standard laboratory conditions: 12∶12 light:dark schedule, standard food (yeast, sucrose, dark corn syrup, molasses, and agar), 25°C and 50% humidity. Wild-type *Canton S* (*CS*) were obtained from the Bloomington Stock Center. *timeless* (*yw*, *tim^01^*) [26] and *cycle* (*cyc^01^*, *ry^506^*) [27] mutants were a gift from Jeff Hall (Brandeis). For experimentation, newly eclosed adult flies were collected under CO_2_ anesthesia and segregated by gender.

#### Drosophila sleep recording

Three-day-old flies were placed into 65 mm glass tubes containing standard lab food and monitored with the Trikinetics activity-monitoring system (Waltham, MA) as previously described [28], [29]. Briefly, activity was recorded in 1 min bins and episodes of quiescence ≥5 min have been empirically defined as sleep. Total sleep time, sleep architecture and sleep homeostasis were calculated using an in-house program according to criteria previously established [28]–[30]. Flies were sleep deprived using the sleep-nullifying apparatus (SNAP) which asymmetrically tilted −60° to +60° such that the sleeping flies were displaced during the downward movement 6 times/minute [28], [29]. Flies were deprived of sleep for 12 h between ZT12 (lights out) to ZT0 (lights on) at which point flies were released into recovery where they remained unperturbed for 48 h. The clock mutants *cyc^01^* and *tim^01^* were maintained and sleep deprived for 7 hrs. under constant darkness. Sleep homeostasis was calculated for each individual as a ratio of the minutes of sleep gained above baseline during the 48 h of recovery divided by the total min of sleep lost during 12 h of sleep deprivation (min gained/min lost).

#### Pharmacology

All drugs were dissolved into standard fly food and flies were manually transferred to tubes with the appropriate pharmacologic agent. After two days of baseline, female flies were placed onto 20 µM paraquat for 16 hours ending at lights on. To differentially induce waking, flies were placed onto either 2.5 mg/mL caffeine or 0.5 mg/mL methamphetamine for 14 hours ending at lights on [9]. Flies were then transferred back to standard food to assess sleep.

#### Quantitative PCR

Total RNA was isolated from 14–20 fly heads by using TRIzol (Invitrogen, Carlsbad, CA) following the manufacturer's protocol. After SD, two-thirds of the flies from each group were frozen, and RNA was extracted from whole heads. The remaining flies (one-third) were monitored for an additional 24 h to assay the size of the homeostatic response. By using this protocol, both gene expression and behavior can be evaluated in siblings that have been exposed to identical environments and experimental manipulations. RNA was treated with RNase-free DNase I (TURBO DNA-free; Ambion, Austin, TX) according to the manufacturer's instructions. Isolated RNA was reverse-transcribed by using SuperScript III (Invitrogen Life Technologies, Carlsbad, CA) according to the manufacturer's instructions. Quantitative PCR using SYBR based technology was performed by using the 7600 Real-Time PCR System (Applied Biosystems, Foster City, CA). A 9-ul aliquot of the cDNA was used in each reaction, and all reactions were performed in duplicate. To minimize cDNA variability between samples, cDNA sets were chosen by matching *RP49* levels. Quantitative values were calculated using the 2-ddCT method [31]. Briefly, CT values were set and then normalized to the control gene, *RP49*, within the respective sample. Subsequently, normalized values were compared between samples.

## Results

### IL-6 levels are increased following Sleep deprivation in rats and humans

**Figure 2:**
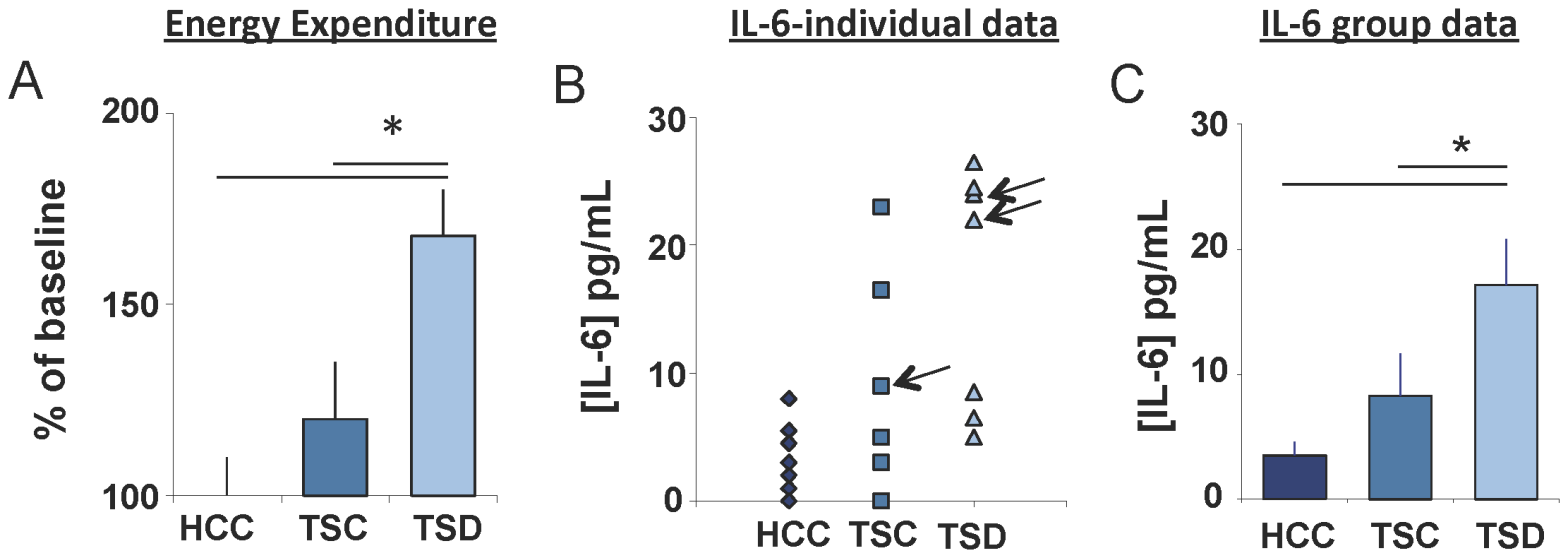
IL-6 is elevated in serum of chronic total sleep deprived rats. (**A**) Energy Expenditure expressed as % of home cage controls (HCC, n = 7) is significantly increased in total sleep deprived (TSD, n = 7) rats compared to yoked controls (TSC, n = 6); One way ANOVA F_(2,11)_ = 6.79, p = .012, * modified Bonferroni test p<0.05 (**B**) Serum concentrations of IL-6 protein from individual TSD, TSC, and HCC rats. Arrows denote which animals tested positive for bacteria. (**C**) Mean concentration of IL-6 from individual rats in (B). One way ANOVA F_(2,17)_ = 5.22, p = .017 * p<0.05 modified Bonferroni test [58]. Data are presented as mean±SEM.

Recent studies have shown that sleep disruption in humans produces enhanced inflammatory signaling as evidenced by increases in serum interleukin-1β (IL-1β), interleukin-6 (IL-6), tumor necrosis factor-alpha (TNF-α), and C-reactive protein (CRP) to name a few [32], [33]. To determine if serum levels of cytokines are also changed in rats in response to sleep deprivation, we evaluated serum levels of TNF α, IL-1β, and INFγ and IL-6 following 8 days of total sleep deprivation using the disk-over-water method. During sleep deprivation, TSD rats lost an average of 72% of their daily NREM and 96% of their daily REM sleep. In contrast, TSC rats lost only 20% of their daily NREM and 47% of their daily REM sleep. Furthermore, TSD rats lost more weight and increased food intake to a larger degree than TSC rats (Figure S1 A,B) such that TSD rats exhibited a significant increase in Energy Expenditure (EE) (Figure 2 A). Consistent with previous reports [34] bacteria was present in the blood of 2 of 7 TSD rats (Rat 1-*Pseudonomas aerguinosa* and *Streptocomyces agalactiae*; Rat 2-*staph Sciuri/lentus*, *strep equisinailes*, *corynebacterium minaticsimum*), 1 of 6 TSC rats (*staph hyicus*, *enterococaus sp*.), and 0 of 7 HCC rats. There was no apparent effect of the presence of bacteria on weight, food intake, or EE. Of the cytokines evaluated, only IL-6 was significantly increased following 8 days of TSD (Figure 2B,C). We found no statistical differences in serum levels of TNF-α, IL-1β, and INF-γ (Figure S2). Thus, chronic total sleep deprivation increases the pro-inflammatory cytokine IL-6 (although see [35]).

**Figure 3:**
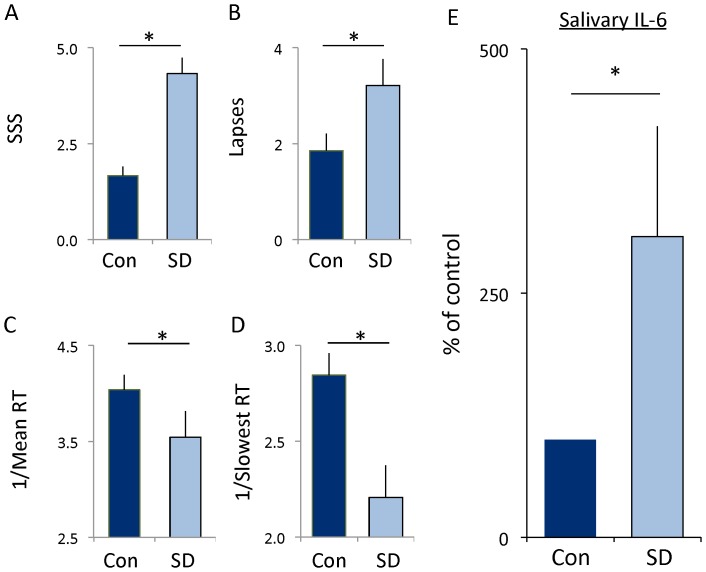
Human subjects show an increase in salivary IL-6. (**A**) Subjective sleepiness as assessed using the Stanford Sleepiness Scale was increased following 30 h of wakefulness. Each subject served as their untreated circadian-matched control; t-test p = 0.0008. (**B**–**D**) Cognitive performance, assessed using the psychomotor vigilance task (PVT), was impaired following 30 h of wakefulness: (**B**) Lapses increased during waking compared to controls; t-test, p = 0.03. (**C**) Mean reaction time, expressed as 1/RT) slowed; t-test p = 0.001. (**D**) Reaction times in the slowest 10% also slowed t-test p = 0002. (**E**) Levels of IL-6 protein from human saliva after 30 hours of waking. Levels in sleep deprived subjects (n = 16) are expressed as a % of that their own untreated circadian-matched sample (con); t-test p = 0.042.

While human studies have found that IL-6 levels are increased in serum following sleep deprivation, sleep restriction and insomnia [36]–[41], it remains unclear whether salivary IL-6 levels are modified by sleep disruption. Given that saliva is a readily accessible biofluid that can be assayed using non-invasive procedures we examined the effects of a night of sleep loss on salivary IL-6 after 30 h of wakefulness. Subjects were admitted to the laboratory at 8 am and were administered the Stanford Sleepiness Scale (SSS), a 10-min version of the psychomotor vigilance task (PVT) and provided a saliva sample. Subjects completed the SSS and the PVT and gave saliva samples every 6 h until 2 pm the following day. As seen in Figure 3A, subjective sleepiness was significantly increased following 30 h of waking. Moreover, sleep deprived subjects displayed an increase in the number of lapses and their reaction times were significantly slower (Figure 3B–D). Thus, our sleep deprivation protocol increased perceived sleepiness and decreased reaction time performance. IL-6 levels were increased following 30 h of sustained waking compared to untreated circadian-matched controls (Figure 3E). These data are consistent with previous observations that IL-6 levels are increased in serum following sleep loss and suggests salivary IL-6 may be an appropriate tool to elucidate the relationship between sleep disruption, perceived sleepiness and IL-6 activation.

### Sleep loss activates inflammatory signaling in flies and humans

**Figure 4:**
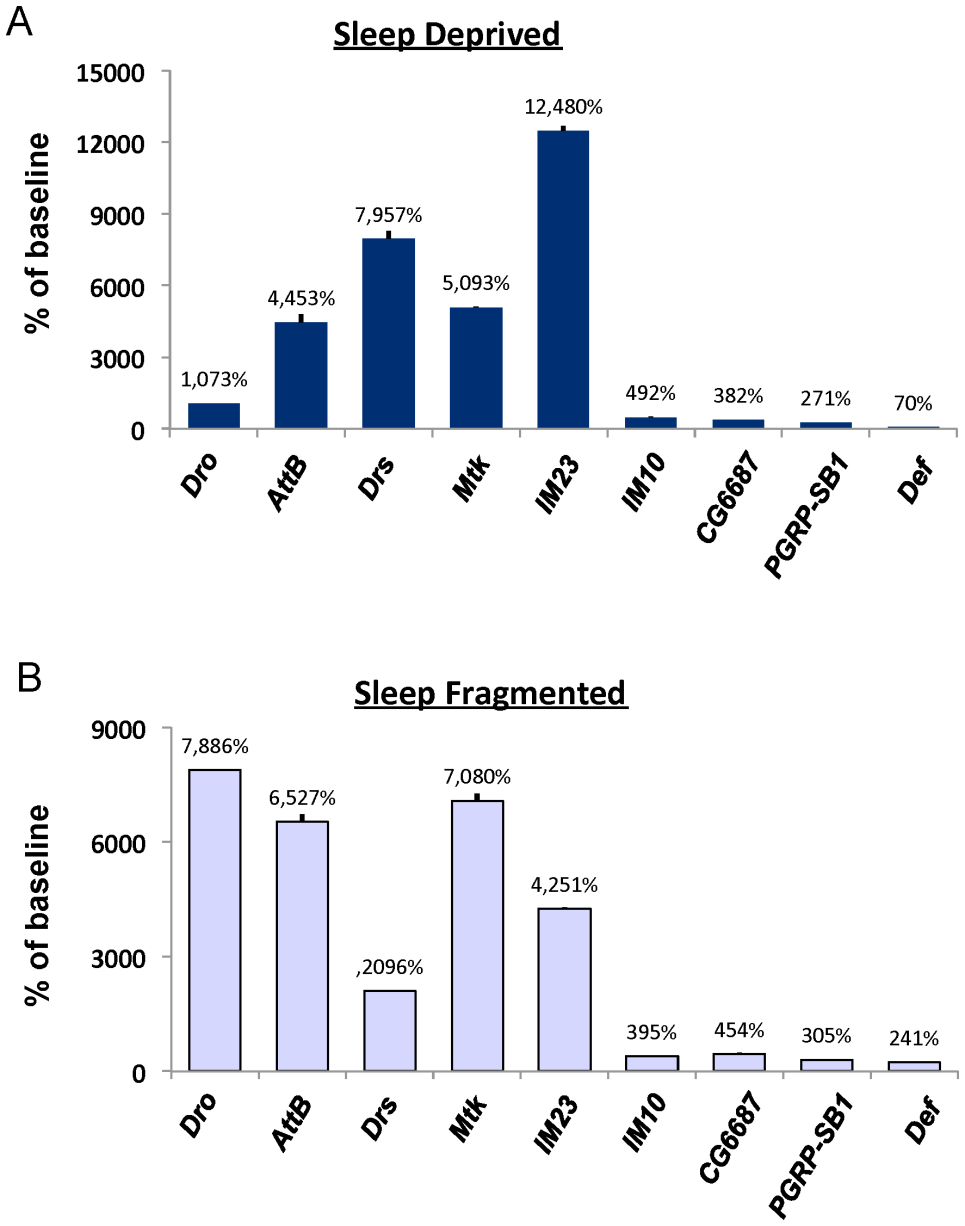
Transcripts for immune-related genes are elevated in flies under conditions of increased sleepiness. (**A**) Immune-related transcripts are significantly elevated in *Cs* flies following a night of sleep deprivation; data are expressed as % of untreated circadian-matched siblings. (**B**) *Cs* flies with normal total sleep time but spontaneously fragmented sleep (average night sleep bouts <30 min) exhibit an increase in immune-related transcripts expressed as % change from siblings with consolidated sleep and matched by total sleep time; error bars are present but not visible given the magnitude of the changes. Abbreviations: *Drosocin* (*Dro*), *Attacin-B* (*AttB*), *Drosomycin* (*Drs*), *Metchnikowin* (*Mtk*), *Immune induced molecule 23* (*IM23*), and *Defensin* (*Def*).

Microarray studies have reported that sleep deprivation increases transcript levels of genes involved in immunity [12], [18], [42]. However, the extent to which immune-related genes are associated with increased sleep-drive/sleepiness remains unknown. In flies, increased sleep drive/sleepiness is operationally defined as the presence of sleep homeostasis following extended waking [9]. As seen in Figure 4A and Table S1, 12 h of sleep deprivation alters mRNA levels of several genes associated with the immune response; some by as much as 80–130 fold. To determine whether the increase in immune-related transcripts was due to sleep loss *per se*, or if these genes were non-specifically activated by the methods used to keep the animals awake (e.g. mechanical perturbation), we profiled immune-related genes in flies that spontaneously exhibit fragmented sleep. We have previously shown that a sub-population (∼8%) of *Cs* flies spontaneously exhibit fragmented sleep, as defined by nighttime sleep bout durations less than 30-min, but obtain normal amounts of total sleep [43]. Despite obtaining the same amount of total sleep as their consolidated siblings, these sleep-fragmented flies behave similarly to sleep deprived flies as measured by deficits in dopamine signaling, increases in the biomarker of sleepiness, *Amylase*, and impaired short-term memory [43]. As seen in Figure 4B and Table S1, in the absence of mechanical perturbation, spontaneously sleep-fragmented flies show similar increases in immune-related genes to that observed in sleep-deprived flies. Importantly, when flies were subjected to oxidative stress, by feeding them paraquat, immune related transcripts were not consistently elevated (Table S1 and Figure S3). Taken together, these data demonstrate that the increases in immune-related transcripts that are observed following sleep deprivation are not due to a general stress response.

**Figure 5:**
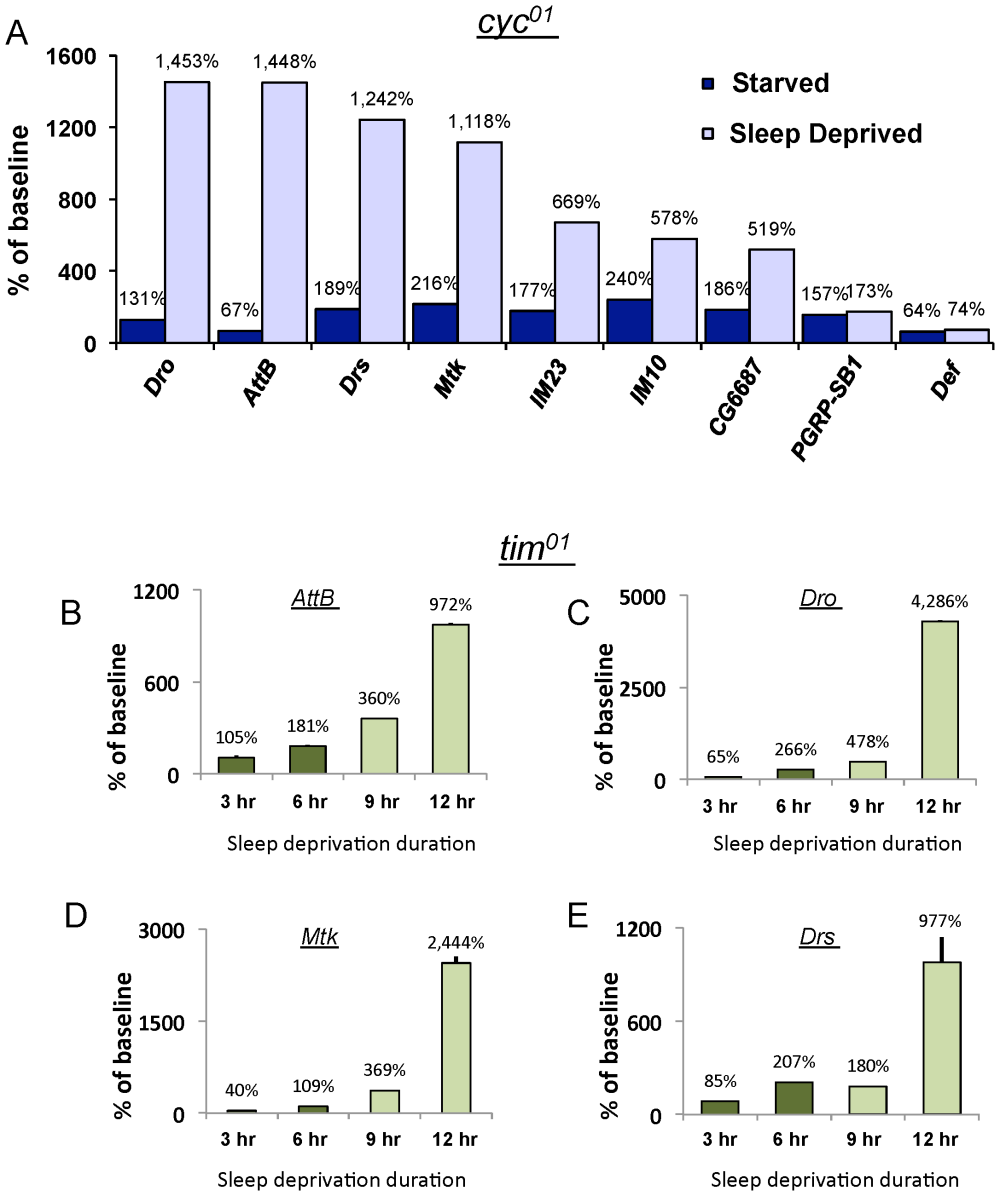
Immune-related transcripts are associated with increased sleep drive in *Drosophila* clock mutants. (**A**) Immune related transcripts are substantially increased in *cyc^01^* mutants following 7 h of sleep deprivation but are not consistently activated when 7 h of waking is induced by starvation; data are expressed as % change from untreated siblings. Experiments were conducted in constant darkness (DD). (**B**–**E**) Transcript levels for *AttB*, *Dro*, *Mtk* and *Drs* in *tim^01^* mutants following 3, 6, 9 and 12 h of sleep deprivation. Immune related transcripts are consistently elevated following 9 and 12 hour sleep deprivation (Light Green) but not after 3 or 6 h of sleep deprivation when no sleep rebound is observed (dark Green).

To evaluate the extent to which immune-related genes are associated with increased sleep drive, rather than simply being elevated during extended waking, we exposed the circadian clock mutant, *cycle* (*cyc^01^*), with either 7 h of sleep deprivation or 7 h of starvation. *cyc^01^* mutants are extremely sensitive to sleep loss and exhibit a sleep rebound that is 10 times greater than wild-type flies even following brief periods of sleep deprivation [29]. However, episodes of waking induced by starvation are not accompanied by 1) a sleep rebound, 2) increases in *Amylase* or 3) deficits in short-term memory [44], [45]. Thus, if immune-related transcripts are indeed biomarkers of sleepiness, they should be activated by sleep deprivation but not by starvation. As seen in Figure 5A and Table S2, several immune related genes are altered when waking is induced by sleep deprivation but not when waking is induced by starvation. In contrast to *cyc^01^*, *timeless* mutants (*tim^01^*) do not display a sleep rebound following 3 h and 6 h of sleep deprivation but show clear signs of sleep homeostasis following 9 and 12 h of sleep deprivation [9], [29], [46]. Thus if immune-related transcripts are associated with periods of waking that are accompanied by elevated sleep drive, we would predict that immune-related transcripts would only be elevated in *tim^01^* mutants following 9 and 12 h of sleep deprivation. As seen in Figure 5B and Table S2, many of the immune genes, including *Attacin-B* (*AttB*), *Metchnikowin* (*Mtk*), *Drosocin* (*Dro*) and *Drosomycin* (*Drs*) followed the expected stepwise increase of expression in *tim^01^* mutants following 9 and 12 h of sleep loss. Thus, the increase in immune-related transcripts is associated with sleepiness and not simply wake time.

**Figure 6:**
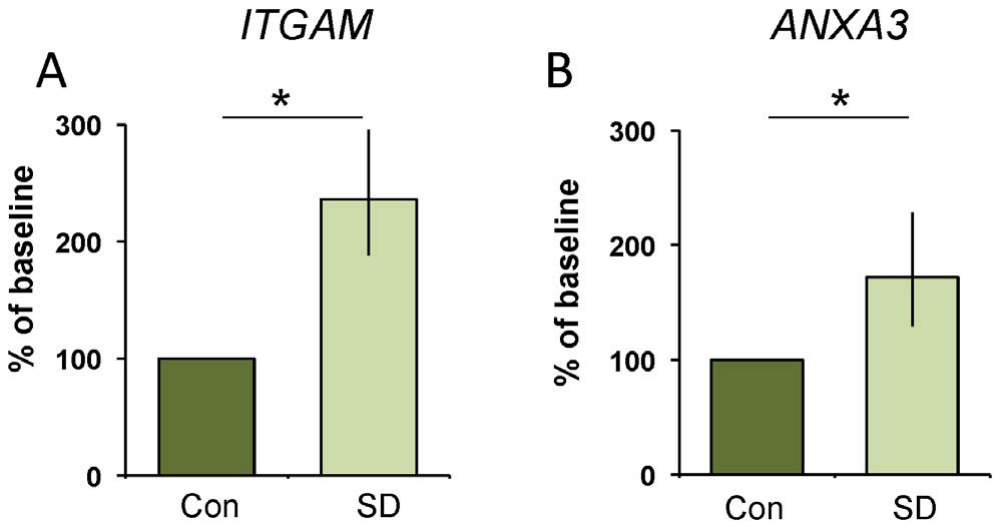
Human Inflammation Low Density Arrays identify genes that are increased following 30 h of waking. (**A**) Salivary transcript levels for *Integrin αM* (*ITGAM*) and (**B**) *Annexin* (*ANXA3*) are increased following 30 hours of wakefulness; t-test, p = .04 and p = .04, respectively. Each subject serves as their own untreated circadian matched control; data are expressed as a percent change from control.

**Figure 7:**
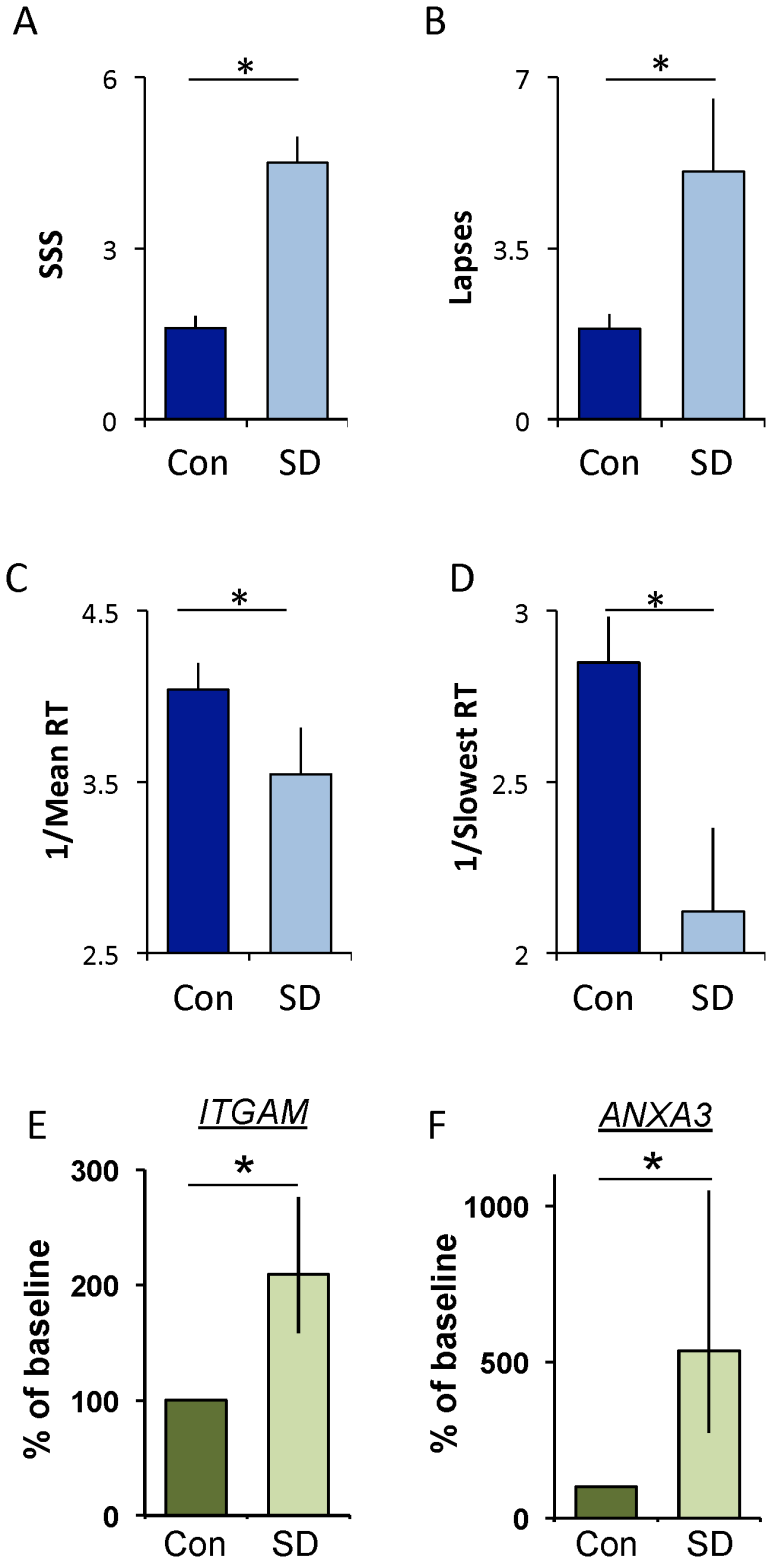
Twenty-four hours of waking increases salivary *ITGAM* and *ANXA3*. (**A**) Subjective sleepiness (SSS) is increased following 24 h of wakefulness. Each subject served as their untreated circadian-matched control; t-test p = 0.0003. (**B**) Lapses increased during waking compared to controls; t-test p = 0.028. (**C**) Mean reaction time, expressed as 1/RT, slowed; t-test p = 0.003 (**D**) Reaction times in the slowest 10% also slowed; t-test p = 0.016. (**E**) Salivary transcript levels for *Integrin αM* (*ITGAM*) and (**F**) *Annexin* (*ANXA3*) are increased following 24 hours of waking; t-test, p = 0.04 and p = 0.04, respectively; data are expressed as a percent change from control.

As mentioned above, an effective test of sleep loss in humans will likely require a panel of biomarkers that 1) respond to increased wake time and 2) have different spectrums of outside influences. Given that immune-related genes appear to be tightly associated with increased sleepiness in flies and that sleep disruption increases serum inflammatory markers in humans, we conducted an unbiased screen of 96 inflammation-related genes using Human Taqman Low Density Arrays. Saliva samples from the subjects in Figure 3 were evaluated at 2 pm under baseline and at 2 pm after 30 h of waking such that each subject served as their own untreated circadian-matched control. Salivary mRNA for one subject was substantially degraded and was excluded from further analysis. We predicted that since the number of transcripts that can be found in saliva is relatively small [16], only a subset of the genes on the Low Density Array would be present in human saliva. Indeed, only 18/96 genes were present in 6 or more of the 7 subjects (Table S3). Interestingly, *Anaxin 3* (*ANXA3*) and *Integrin αM* (*ITGAM*) were significantly elevated following 30 h of waking (Figure 6 A,B). Because this was a screen to identify new candidate biomarkers we chose not to correct for multiple comparisons in the initial analysis. However, as an alternative to making a correction for multiple comparisons, we conducted additional tests to determine if the changes in ANXA3 and ITGAM were reproducible. We designed new primers for each gene and evaluated their transcript levels using Syber Green, a different technology than the Taqman based Low Density Array. We then evaluated independent saliva samples from each subject after 24 h of waking. As seen in Figure 7A–D, subjects reported feeling sleepy and had significantly more lapses and slower reaction times on the PVT after 24 h of waking. Importantly, both *ANXA3* and *ITGAM* were significantly increased after 24 hours of sleep deprivation (Figure 7E–F). Thus, transcripts for *ANXA3* and *ITGAM* are elevated in human saliva during sleep loss.

**Figure 8:**
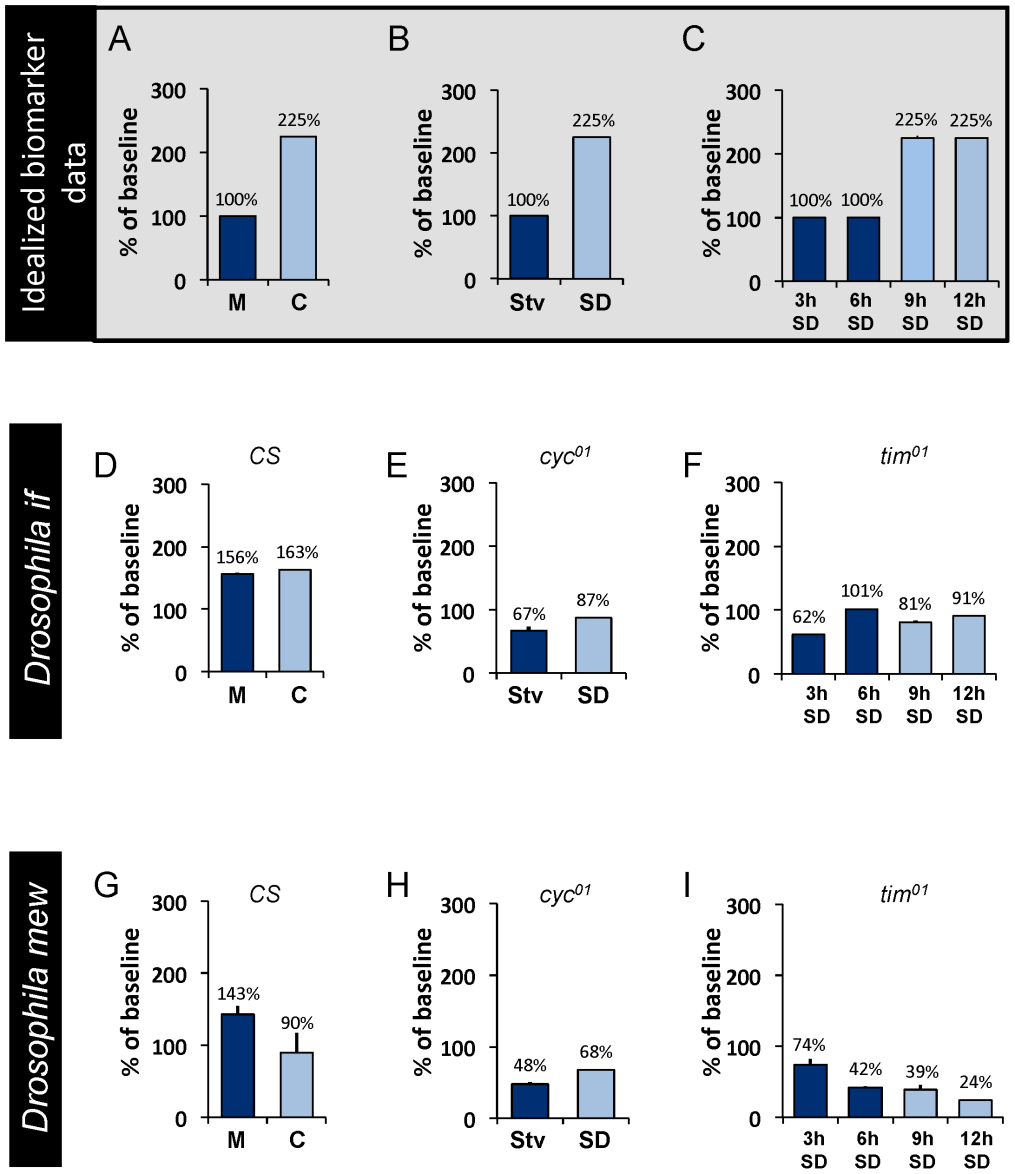
*Drosophila* homologues of *ITGAM*, *If* and *mew* do not behave as biomarkers in flies. (**A**–**C**) A cartoon of idealized data of expected transcriptional patterns exhibited by biomarkers of sleepiness for comparison to empirical data in D–I. **D, G**) Transcripts for *if* and *mew* are not elevated in *Cs* flies when waking is induced either by caffeine (C) (which is accompanied by a sleep rebound) or methamphetamine (M) (which is not followed by an increase in sleep) compared to untreated siblings. (**E,H**) *cyc^01^* mutants do not show an increase in *if* or *mew* transcripts following sleep deprivation. (**F,I**) There is no step increase in if or mew transcripts following 6 or 9 h of sleep deprivation in *tim^01^* mutants.

**Figure 9:**
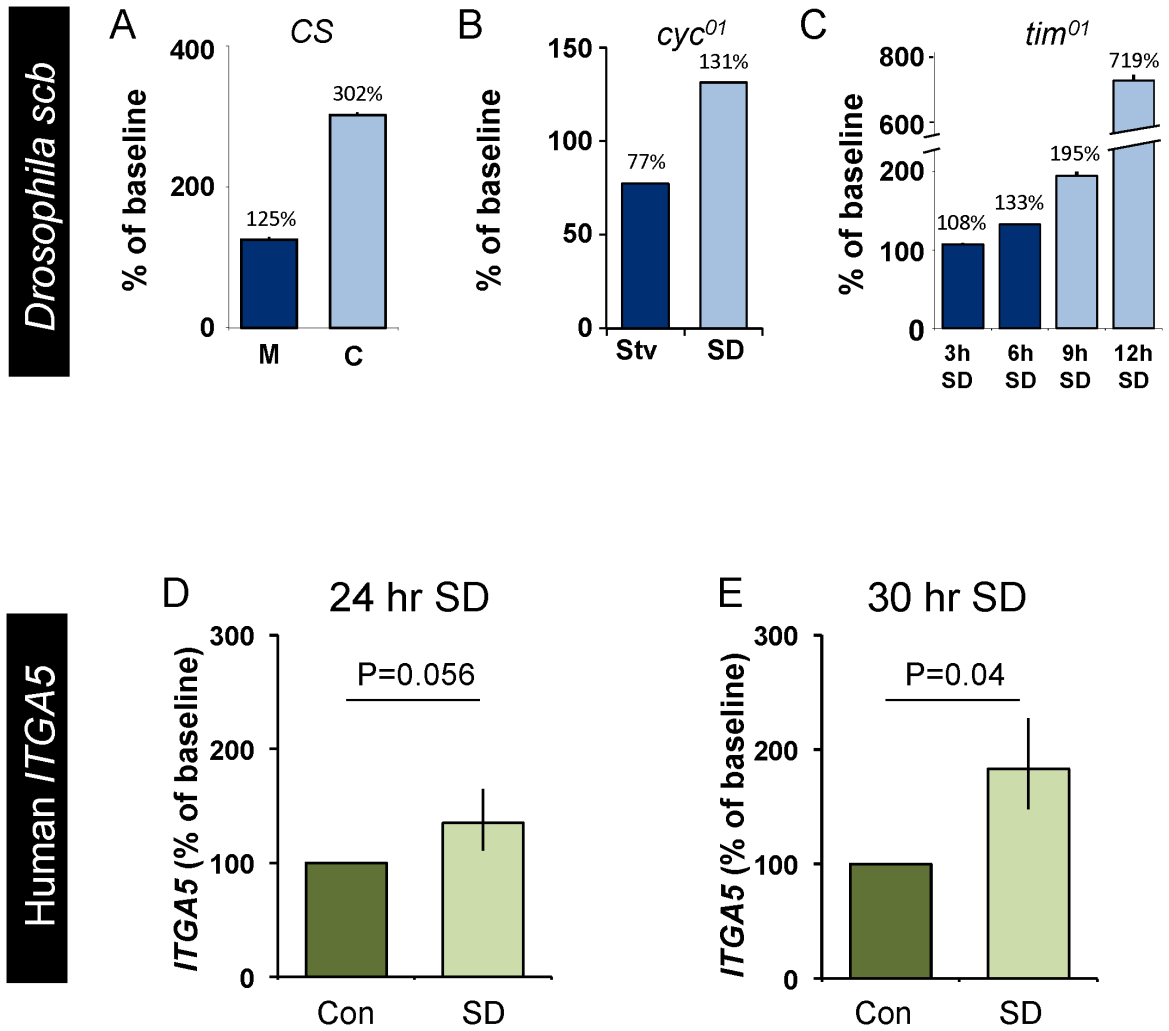
The *Drosophila* integrin *scb* and Human *ITGA5* are modified by sleep loss. (**A**) *scb* is elevated following caffeine administration but not after waking induced by methamphetamine. (**B**) *scb* is elevated in sleep deprived *cyc^01^* mutants but is not increased when waking is induced by starvation. (**C**) *scb* mRNA levels remain low following deprivations that do not activate homeostatic mechanisms (3 and 6 h SD), but are elevated following deprivations that activate homeostatic mechanisms (9 and 12 h SD). (**D**–**E**) Salivary *ITGA5* transcripts are increased in humans following 30 h of wakefulness while 24 h of wakefulness do not quite reach significance; t-test and p = 0.04 and p = 0.056 respectively. Each subject serves as their own circadian matched untreated control; data are expressed as a percent change from control.

Given that the sample size of the human sleep deprivation study is relatively small, we re-turned to the fly to determine whether transcripts of the *Drosophila* homologues of *ITGAM* would behave as biomarkers of sleepiness. As seen in Figure 8, the transcript levels of two closely related homologs (http://eugenes.org/), *multiple edematous wings* (*mew*) and *inflated* (*if*), did not behave as a biomarkers when evaluated using the paradigms used to validate *Amylase* and immune-related genes described above (Figure 5). For comparison, we present a cartoon of idealized data depicting the pattern of expression of a biomarker of sleepiness in Figure 8 A–C. Although this outcome was disappointing, it widely recognized that under some circumstances genes can display complementary expression profiles in flies and mammals. For example, in *Drosophila*, transcripts for the circadian-clock gene *Clock* exhibit a circadian pattern of expression while transcripts for *cycle* are stable across the circadian day; in contrast transcripts for the mammalian homologue of *cycle*, *Bmal*, exhibit a circadian pattern while *CLOCK* transcript levels remain stable. This observation suggested to us that, in the fly, alternate members of the integrin family might be responsive to sleep loss. Therefore, we also examined the expression profiles of *scab* (*scb*), *αPS5*, *αPS4*, *myospheriod*, and *βν integrin* using protocols that can disassociate wake time from sleep drive [9]. As seen in Figure 9 and Table S4, only transcript levels of *scb* were associated with elevated sleepiness. That is, in *cyc^01^* mutants, *scb* transcripts were significantly increased following 7 h of sleep deprivation but were not changed when 7 h of waking was induced by starvation. Since previous studies have shown that a sleep rebound is observed following waking induced by caffeine but not waking induced by methamphetamine administration [9], [28], [46], [47], we evaluated *scb* mRNA in wild-type flies fed caffeine and methamphetamine. As seen in Figure 9B, *scb* transcript levels were significantly increased when waking was induced by caffeine but not when waking was induced by methamphetamine. Finally, we evaluated *scb* in *tim^01^* flies after 3, 6, 9, and 12 hours of sleep deprivation. *scb* displayed a stepwise increase at 9 and 12 hours of sleep deprivation (Fig 9C and Table S5). Together these data indicate that while the *Drosophila* homologues of ITGAM, *mew* and *if*, do not behave as biomarkers, *scb* is consistently increased following waking episodes that are accompanied by a sleep rebound. Interestingly, a human homologue of *scb* (http://eugenes.org/), *Integrin α5* (*ITGA5*), is not included on the Taqman Low Density Array. Thus, we asked whether salivary transcripts of *ITGA5* would be responsive to 24 h and 30 h of waking. As seen in Figure 9E, *ITGA5* transcription was significantly elevated at 30 h. Together, these data indicate that fly and human discovery experiments are mutually reinforcing and can be used interchangeably to identify candidate biomarkers of sleep loss.

## Discussion

Given the burden that inadequate sleep and sleep disorders place on society, there is consensus of the need for a simple, inexpensive and objective measure of sleep loss [8]. Although assays based upon electrophysiological measurements, pupillometry and several behavioral tests, most notably the psychomotor vigilance test, have shown promise, these methods have been difficult to transfer into the field and into occupational settings where they might have the largest impact on public health and safety [5], [8]. We have hypothesized that as a rich and easily accessible biofluid, saliva can be mined to identify analytes that are responsive to sleep loss [13]. Moreover, since we expect that an effective test of sleep loss will likely be composed of a panel of multiple biomarkers, it is important to identify and validate a number of candidates that have a different spectrum of outside influences. Thus, a major goal of this study was to demonstrate the feasibility of conducting an unbiased screen using saliva to identify analytes that respond to sleep deprivation. Since the relationship between sleep and the immune system has been the topic of intensive investigations for over 25 years [17], we began by interrogating the salivary transcriptome to determine whether immune related genes are modified by sleep loss. We then exploited the evolutionary conservation of sleep to further characterize the behavior of candidate biomarkers using *Drosophila* genetics. Our data indicate that IL-6, *ITGAM*, *ANAX3*, and *ITGA5* are candidates for inclusion in a panel of salivary biomarkers of sleep loss in humans.

We became interested in examining the effects of sleep deprivation on salivary IL-6 based upon previous observations that serum inflammatory markers are strongly modulated by sleep disruption in humans [19], [20], [36]. Indeed, over the last decade studies have consistently reported that sleep disruption is associated with changes in serum IL-6 [48]. For example, 2 h of sleep restriction in young healthy subjects for 1 week results in an increase in plasma IL-6 levels in the morning and afternoon [39]. In addition, poor quality sleep enhances the daytime secretion of IL-6 [49]. Interestingly, serum IL-6 levels are negatively correlated with sleep quality and have also been associated with daytime somnolence and fatigue [19], [40], [50]. Thus, inflammatory markers in saliva, including IL-6, are viable candidates for monitoring sleep disruption.

In the current study, we found that the increase in plasma IL-6 is also seen in rats following 8 days of chronic total sleep deprivation. Interestingly, long-term sleep deprivation appears to leave rats more susceptible to infection [51]. Nonetheless, it is unlikely that bacteremia resulted in immune activation and IL-6 induction as only 2 of 7 rats showed bacteremia after 8 days of chronic total sleep deprivation. One limitation of the rodent study was that we were only able to sample IL-6 levels at one time point. Given that IL-6 levels might change dynamically over time, a more extensive time-course will be needed to determine when IL-6 levels are increased and whether they remain elevated during chronic sleep loss. It is interesting to note that in the fly, there is also an upregulation of immune genes with sleep deprivation [18]. Indeed, we extend these results to show that sleep fragmentation is capable of inducing immune genes in the fly without the need for mechanical perturbation to keep the animals awake. Similarly, immune genes are not consistently activated by exposure to oxidative stress. Importantly, we found that waking induced by starvation, which is not associated with a sleep rebound or cognitive deficits in flies [44], does not activate immune genes. Thus, immune activation may be may be a critical component in the response to sleep deprivation, since it is observed in rats, flies, and humans. Moreover, our data suggest that immune activation is a physiologic response to sleep loss and not necessarily a secondary response to infection or stress.

Our data demonstrate that salivary IL-6 is increased in humans following sleep loss and is consistent with studies measuring serum IL-6. Since saliva is an easily accessible biofluid, salivary IL-6 may eventually become useful as an additional quantitative tool for assessing sleep loss. With this in mind, it is important to note that both serum and salivary IL-6 are influenced by circadian rhythms [49], [52]. The circadian influence on IL-6 has two implications. First, salivary IL-6 may not be equally effective at detecting sleep loss at all circadian times. Although this may interfere with using salivary IL-6 in the field, IL-6 can still be used in the laboratory where circadian factors can be more precisely controlled [e.g. phenotyping human subjects with polymorphisms in genes that influence sensitivity to sleep loss [10], [53] or in subjects with differential cognitive impairments following sleep loss [54]]. Secondly, the prevalence of analytes that are influenced by circadian rhythms suggests that it may be necessary to generate separate panels of biomarkers that are optimized for specific circadian times. Although this may seem less than ideal, it may expedite the evaluation of biological tests of sleep loss that can be used in real-world settings. In any event, salivary IL-6 is increased following 30 h of waking and may prove to be a simple, accessible and inexpensive marker to assess sleep loss in humans under a variety of experimental settings.

In previous studies we have used the fly as a starting point to identify genes that might also change their expression profile in human saliva following sleep deprivation [9], [11], [12]. In the current study, candidate genes were identified first in humans and then, using a cross-translational approach, validated using *Drosophila* genetics. It is exciting to note that several recent papers have used the fly to validate the role of genes that were first identified in humans [55]–[57]. Together with these studies our data demonstrate that *Drosophila* can be used as a model organism to quickly and efficiently understand the physiologic and behavioral implication of genes that have been identified in larger screens in humans. Indeed, a cross-translational approach reinforces, once again, the evolutionary conservation of sleep regulatory mechanisms.

One potential weakness of this study is the low number of human subjects. To ensure that our results are not an artifact of sampling, we have taken several approaches to validate our initial findings that *ITGAM* is upregulated in the saliva of humans sleep deprived for 30 hours. The first was articulated above showing that homologous genes respond similarly in *Drosophila*. The second approach was to confirm that the same transcripts were elevated in an independent sample collected after 24 hours of wakefulness using a different strategy to quantify transcripts. Even with our low sample number, transcripts for *ITGAM* and *ANXA3* were elevated at both time points using different techniques. Interestingly, transcript levels of *ITGA5*, which were not on the Taqman Low Density Array but were included based upon the behavior of *Drosophila* transcripts, were elevated at 30 h and just missed being significantly elevated (p = 0.056) at 24 h waking. Together, our data demonstrate that inflammatory genes including ITGAM, ANAX3, and ITGA5 are candidates for inclusion in a panel of biomarkers of sleep loss in humans.

## Supporting Information

Figure S1 (**A**) Mean Daily food intake and (**B**) Body weight in TSD and TSC rats expressed as percentage of baseline. *p<0.05; Data are presented as mean ± SEM.
(TIFF)

Figure S2 (**A**) tumor necrosis factor α(TNFα), (**B**) interferon γ (INFγ), and (**C**) interlukein-1 β (IL-1β) were not different after sleep deprivation in rats that have been sleep deprived (TSD), their yoked controls (TSC) and home-cage controls (HCC). One way ANOVA F_(2,17)_ = 1.016, p = .38, One way ANOVA F_(2,17)_ = 1.92, p = .17, One way ANOVA F_(2,17)_ = 0.433, p = .64, respectively.
(TIFF)

Figure S3 **The chemical inducer of stress, paraquat, does not consistently elevate transcript levels of immune genes.** Data are presented as % of untreated controls. *Cs* female flies were placed onto 20 µM paraquat or vehicle for 16 hours ending at lights on (ZT-0). Total RNA was isolated from fly heads and evaluated using Quantitative PCR.
(TIFF)

Table S1 Transcript levels of 21 immune-related genes from the heads of wild-type *Canton S* flies. Cs flies were sleep deprived for 12 hours and compared to untreated, circadian-matched siblings. Sleep fragmented flies with nighttime sleep bouts less than 30 minutes were matched for total sleep time with siblings that exhibited consolidated sleep. Cs flies treated with paraquat were compared to vehicle fed controls. Data presented as fold change ± SEM.
(TIFF)

Table S2 Transcript levels of 21 immune related genes from the heads of *cyc^01^* and *tim^01^* flies. *cyc^01^* flies were sleep deprived ant starved for 7 hrs. and values were compared to unperturbed controls. *tim^01^* flies were sleep deprived for 3, 6, 9, and 12 hours and compared to unperturbed *tim^01^* siblings. All experiments were conducted in DD. Data presented as fold change ± SEM.
(TIFF)

Table S3 Eighteen out of 96 genes from the Human Inflammation Low-Density Array were detected in 6 out of the 7 subjects.
(TIFF)

Table S4 Transcriptional changes of integrins in *Cs* flies. *Cs* flies were sleep deprived for 12 hours and compared to untreated, circadian-matched siblings. Sleep fragmented flies with nighttime sleep bouts less than 30 minutes were matched for total sleep time with siblings that exhibited consolidated sleep. *Cs* female flies were placed onto 20 µM paraquat or vehicle for 16 hours ending at lights on (8 am). Total RNA was isolated from fly heads and evaluated using Quantitative PCR. Data presented as fold change ± SEM.
(TIFF)

Table S5 Transcript levels of integrin related genes from the heads of *cyc^01^* and *tim^01^* flies. *cyc^01^* flies were sleep deprived and starved for 7 hrs. and values were compared to unperturbed controls. *tim^01^* flies were sleep deprived for 3, 6, 9, and 12 hours and compared to unperturbed *tim^01^* siblings. All experiments were conducted in DD. Data presented as fold change ± SEM.
(TIFF)
