# Supplementary figures and images for: Cross-Translational Studies in Human and Drosophila Identify Markers of Sleep Loss
Source: PLoS One. 2013 Apr 24;8(4):e61016. doi: 10.1371/journal.pone.0061016 (PMC3634862; doi:10.1371/journal.pone.0061016)

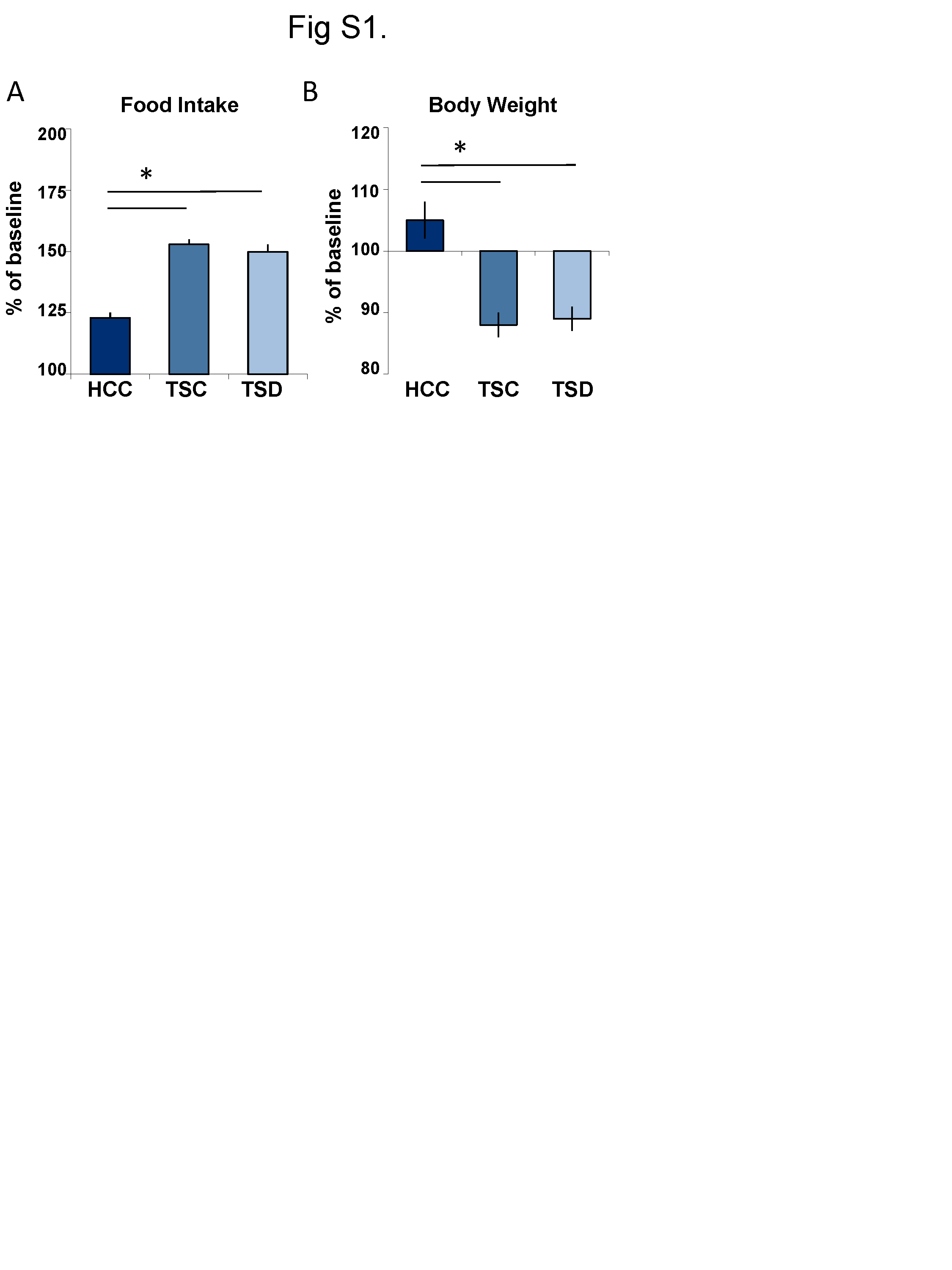

Supplement: Figure S1 — (A) Mean Daily food intake and (B) Body weight in TSD and TSC rats expressed as percentage of baseline. *p<0.05; Data are presented as mean ± SEM. (TIFF) [file pone.0061016.s001.tiff]

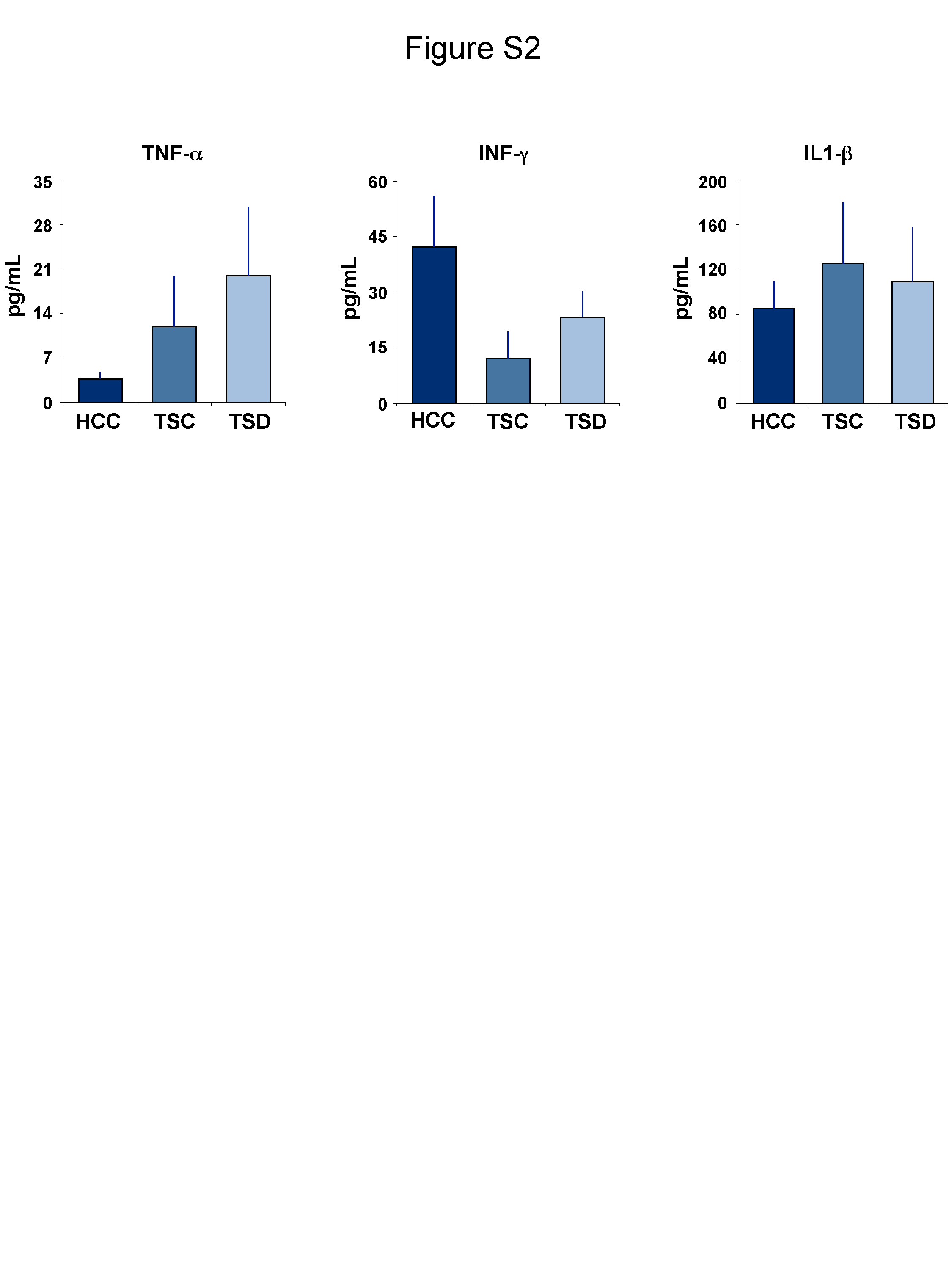

Supplement: Figure S2 — (A) tumor necrosis factor α(TNFα), (B) interferon γ (INFγ), and (C) interlukein-1 β (IL-1β) were not different after sleep deprivation in rats that have been sleep deprived (TSD), their yoked controls (TSC) and home-cage controls (HCC). One way ANOVA F(2,17) = 1.016, p = .38, One way ANOVA F(2,17) = 1.92, p = .17, One way ANOVA F(2,17) = 0.433, p = .64, respectively. (TIFF) [file pone.0061016.s002.tiff]

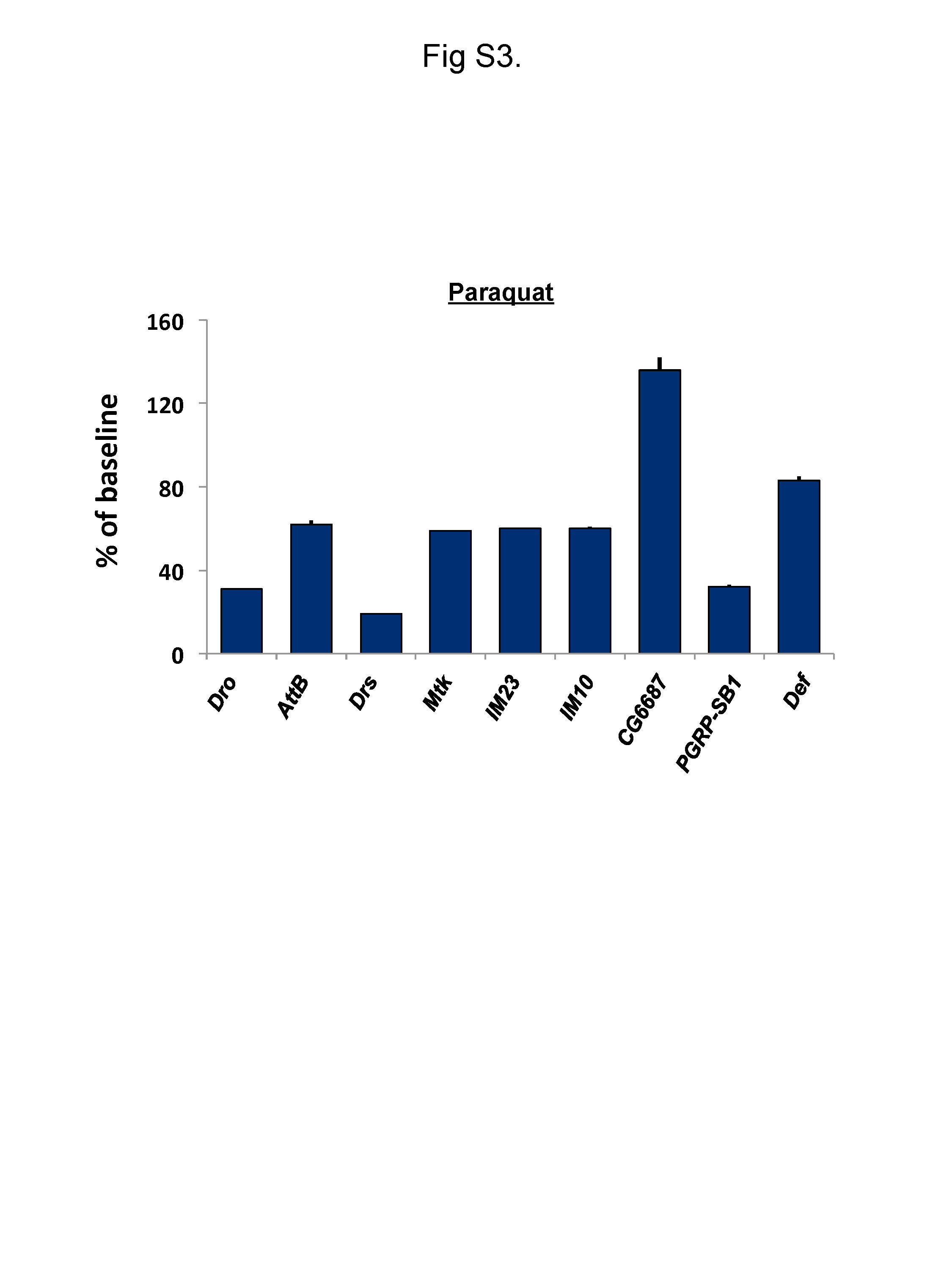

Supplement: Figure S3 — The chemical inducer of stress, paraquat, does not consistently elevate transcript levels of immune genes. Data are presented as % of untreated controls. Cs female flies were placed onto 20 µM paraquat or vehicle for 16 hours ending at lights on (ZT-0). Total RNA was isolated from fly heads and evaluated using Quantitative PCR. (TIFF) [file pone.0061016.s003.tiff]

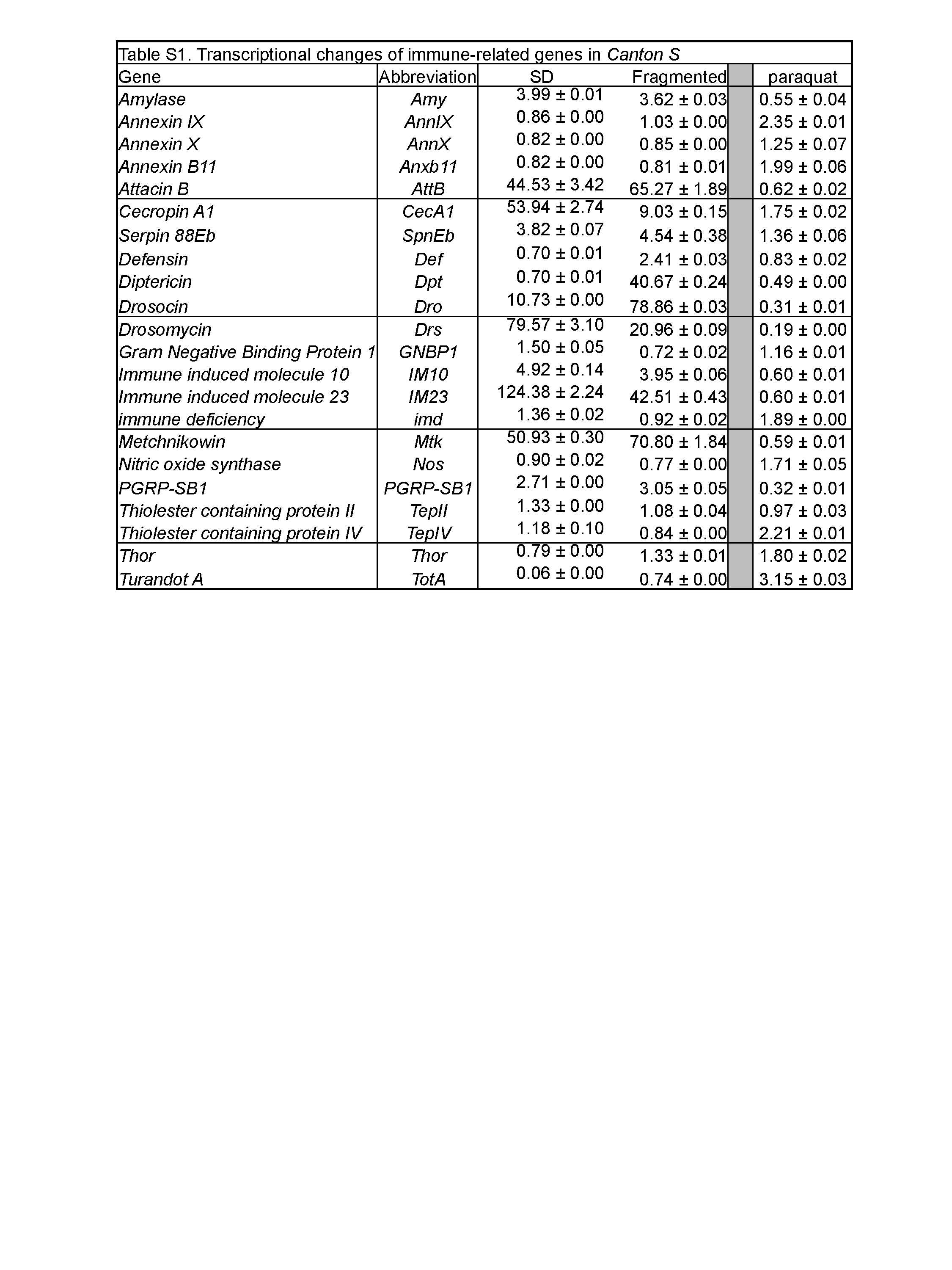

Supplement: Table S1 — Transcript levels of 21 immune-related genes from the heads of wild-type Canton S flies. Cs flies were sleep deprived for 12 hours and compared to untreated, circadian-matched siblings. Sleep fragmented flies with nighttime sleep bouts less than 30 minutes were matched for total sleep time with siblings that exhibited consolidated sleep. Cs flies treated with paraquat were compared to vehicle fed controls. Data presented as fold change ± SEM. (TIFF) [file pone.0061016.s004.tiff]

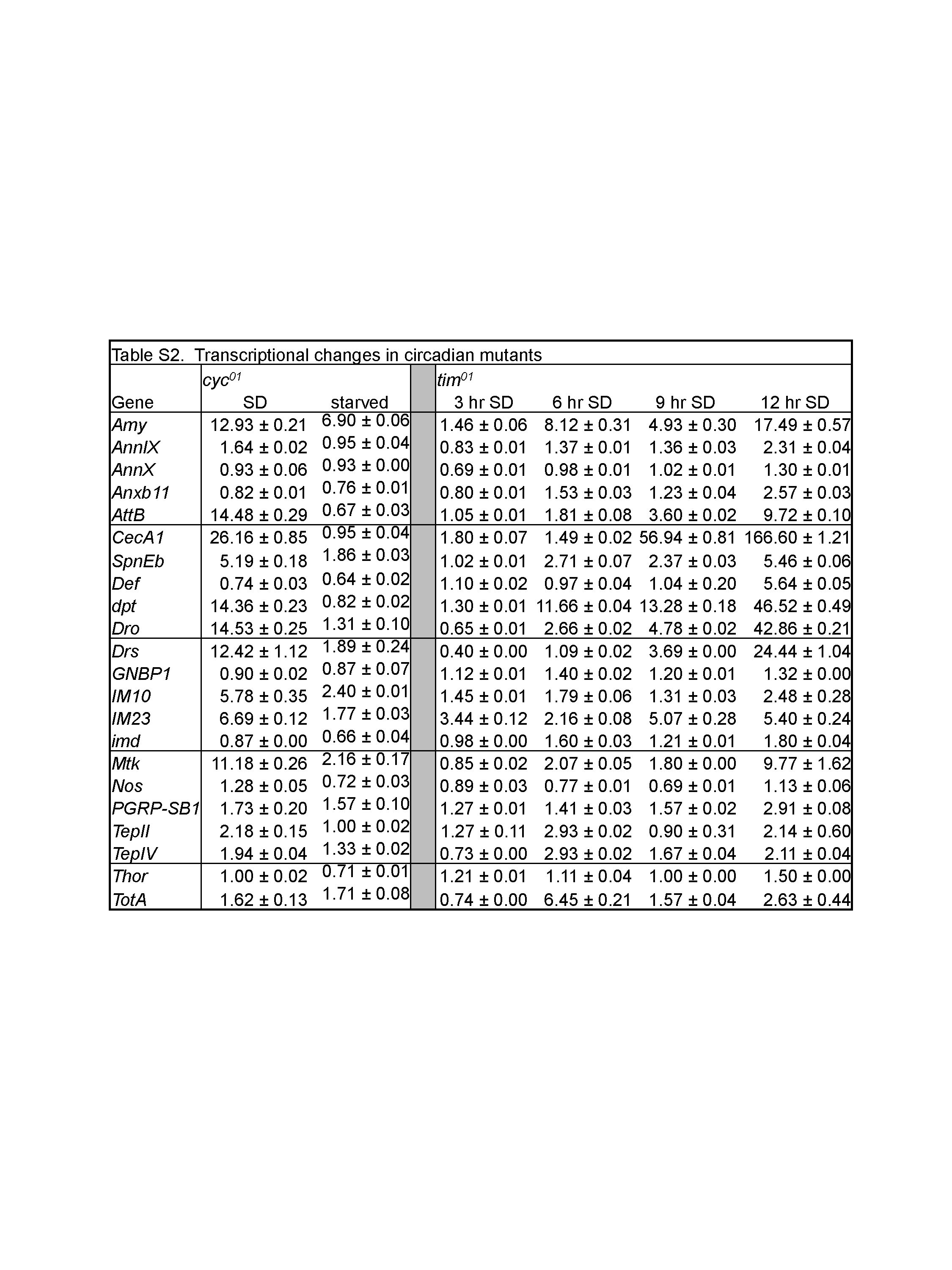

Supplement: Table S2 — Transcript levels of 21 immune related genes from the heads of cyc01 and tim01 flies. cyc01 flies were sleep deprived ant starved for 7 hrs. and values were compared to unperturbed controls. tim01 flies were sleep deprived for 3, 6, 9, and 12 hours and compared to unperturbed tim01 siblings. All experiments were conducted in DD. Data presented as fold change ± SEM. (TIFF) [file pone.0061016.s005.tiff]

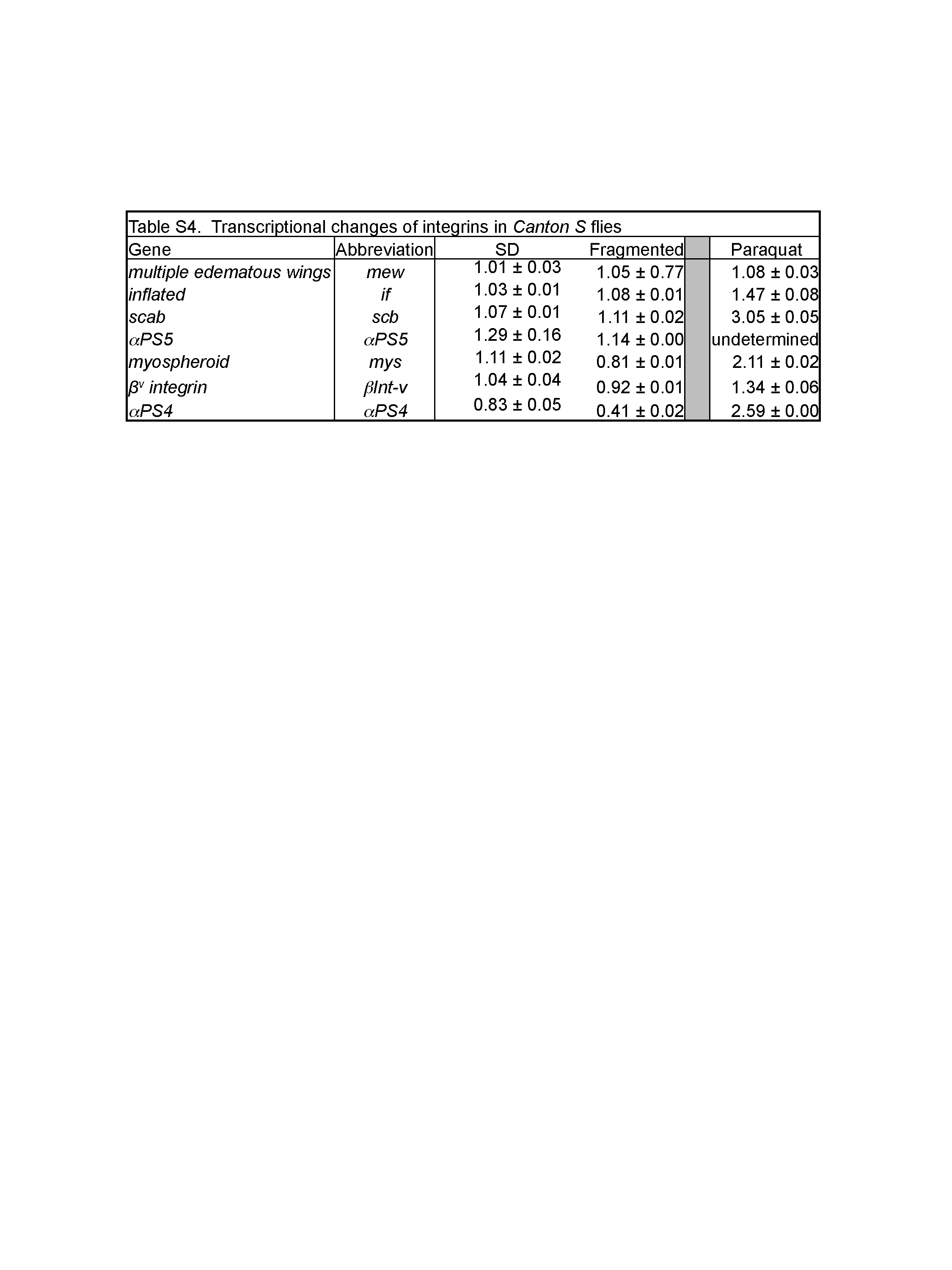

Supplement: Table S4 — Transcriptional changes of integrins in Cs flies. Cs flies were sleep deprived for 12 hours and compared to untreated, circadian-matched siblings. Sleep fragmented flies with nighttime sleep bouts less than 30 minutes were matched for total sleep time with siblings that exhibited consolidated sleep. Cs female flies were placed onto 20 µM paraquat or vehicle for 16 hours ending at lights on (8 am). Total RNA was isolated from fly heads and evaluated using Quantitative PCR. Data presented as fold change ± SEM. (TIFF) [file pone.0061016.s007.tiff]

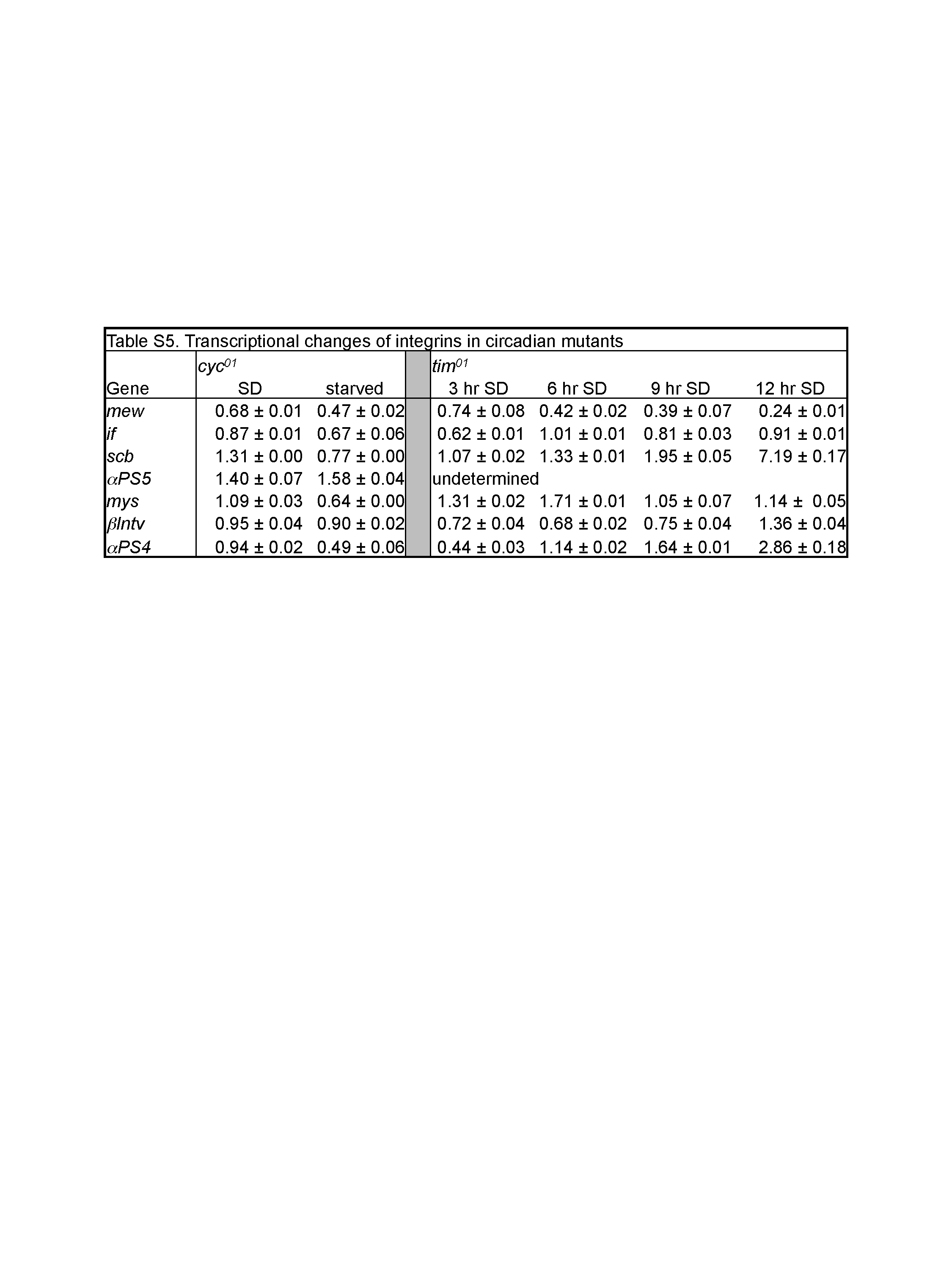

Supplement: Table S5 — Transcript levels of integrin related genes from the heads of cyc01 and tim01 flies. cyc01 flies were sleep deprived and starved for 7 hrs. and values were compared to unperturbed controls. tim01 flies were sleep deprived for 3, 6, 9, and 12 hours and compared to unperturbed tim01 siblings. All experiments were conducted in DD. Data presented as fold change ± SEM. (TIFF) [file pone.0061016.s008.tiff]
